# New Postbiotic Derived from Sequential Fermentation of Two *Lacticaseibacillus* Strains Exerts Beneficial Effects on Epithelial Gut Barrier and Innate Immunity in Human Enterocytes

**DOI:** 10.3390/microorganisms14040931

**Published:** 2026-04-20

**Authors:** Franca Oglio, Alessia Cadavere, Monia De Aloe, Anna Lintura, Marco Michelini, Chiara Luongo, Serena Coppola, Alessandra Agizza, Erika Caldaria, Laura Carucci

**Affiliations:** 1 Department of Translational Medical Science, University of Naples Federico II, 80131 Naples, Italy; alecadavere@icloud.com (A.C.); moniadealoe02@gmail.com (M.D.A.); annalintura03@gmail.com (A.L.); marcom941@hotmail.com (M.M.); chialu86@gmail.com (C.L.); serena.coppola3@unina.it (S.C.); agizza.alessandra@gmail.com (A.A.); erikacaldaria@gmail.com (E.C.); laura.carucci@unina.it (L.C.); 2 ImmunoNutritionLab, CEINGE Biotecnologie Avanzate Franco Salvatore, 80131 Naples, Italy; 3 NutriTechLab Academic Spin-Off, University of Naples Federico II, 80131 Naples, Italy; 4 Task Force for Microbiome Studies, University of Naples Federico II, 80131 Naples, Italy

**Keywords:** postbiotic, cell proliferation, tight junction proteins, occludin, zonula occludens 1, mucus protein Mucin-2, lactase, human beta-defensin 2

## Abstract

The efficacy of postbiotics varies significantly between different strains and preparation processes. We aimed at evaluating the effect of an innovative postbiotic product (iPB) generated through the sequential fermentation of *Lacticaseibacillus rhamnosus* GG and *Lacticaseibacillus paracasei* NPB-01, compared to single-strain postbiotics, on epithelial barrier integrity and innate immunity in human enterocytes using a Caco-2-cell-based experimental model by measuring human enterocyte proliferation and differentiation (lactase expression), tight junction proteins (occludin and zonula occludens 1, ZO-1), and mucus protein Mucin-2 (Muc-2) expression. The modulatory action on the major innate immunity peptide, Human Beta-Defensin 2 (HBD-2), production was also assessed. The iPB exposure resulted in a higher up-regulation of human enterocyte proliferation and differentiation, as suggested by higher lactase expression, and of occludin, ZO-1, and MUC2 expression compared with the single-strain postbiotics, suggesting a beneficial synergistic action in modulating the epithelial gut barrier. Furthermore, iPB induced a higher production of HBD-2, suggesting a synergistic enhancement of innate immune response. Our findings suggested that the sequential fermentation process could act as a biotechnological catalyst, optimizing the gut-barrier-protective properties and the immunomodulatory action of *Lacticaseibacillus* strains. This study introduces iPB as a high-performance postbiotic candidate for the prevention and management of conditions characterized by alterations in epithelial gut barrier and innate immunity.

## 1. Introduction

Postbiotics, which comprise inanimate microorganisms and/or their cellular components and metabolites, are gaining increased attention for their potential health benefits [1]. Although these beneficial actions resemble those of probiotics, the stability and safety of postbiotics make them an appealing alternative, in particular for vulnerable populations, including newborns, elderly people, and patients with clinical states, for whom the use of probiotics may be not recommended [2,3,4]. Emerging findings suggest that the health benefits of selected postbiotics can match, and may even exceed, those reported for their probiotic counterparts [2,5,6]. Extensive research has uncovered that selected postbiotic products can exert beneficial modulatory actions on the microbiome, epithelial barrier, nervous system, and immune and metabolic pathways [5,7,8].

Processes commonly used for producing postbiotics include fermentation and microbial inactivation. The fermentation process could increase the microorganism population and the production of several cellular components and metabolites, all of which can exhibit functional activities [3]. Microbial inactivation can be achieved through various methods, including heat treatment, sonication, high-pressure treatment, and enzymatic treatment [9]. The effectiveness of postbiotics can vary significantly comparing different strains and preparation processes; thus, testing the efficacy of each single postbiotic product is mandatory to ensure that it can deliver the intended health benefits [10]. The majority of available data derives from studies on a single bacterial strain, but it has been suggested that the presence of multiple microorganisms can increase the beneficial health effects compared to single-strain preparations thanks to the synergistic action of different microorganisms [11,12]. However, most of the products are obtained by growing the microorganisms separately and then combining them in the formulation of the finished product or by co-fermenting two or more microorganisms simultaneously [12,13]. Co-fermentation is based on the simultaneous processing of different living microorganisms to take advantage of their metabolic cooperation, which can lead to improved fermentation performance or the development of products with increased beneficial potential. The dark side of this process could be the possible competition between microorganisms [13].

An innovative postbiotic product deriving from the sequential fermentation of two *Lacticaseibacillus* strains, *L. rhamnosus* GG and *L. paracasei* NPB-01, has increasingly been developed with the aim to increase the beneficial modulation of body functions. Here, we comparatively evaluated the beneficial actions exerted by this innovative postbiotic product vs. two of the same single heat-inactivated probiotic strains on epithelial barrier and innate immunity in human enterocytes.

## 2. Materials and Methods

### 2.1. Postbiotic Products

Four different postbiotic products were tested in the study. The first one was obtained by fermenting *L. rhamnosus* GG (ATCC 53103) on MRS broth for 24 h at 37 °C. At the end of the fermentation process, a heat treatment was conducted (90 °C, 1 min) to inactivate the bacterial biomass, yielding the postbiotic preparation (LGGp). The second one was obtained from the fermentation of *L. paracasei* NPB-01 (DSM 34367) on MRS broth for 24 h at 37 °C. At the end of the fermentation process, a heat treatment (90 °C, 1 min) was applied to inactivate the bacterial biomass, obtaining the postbiotic preparation (Lpp).

**Figure 1:**
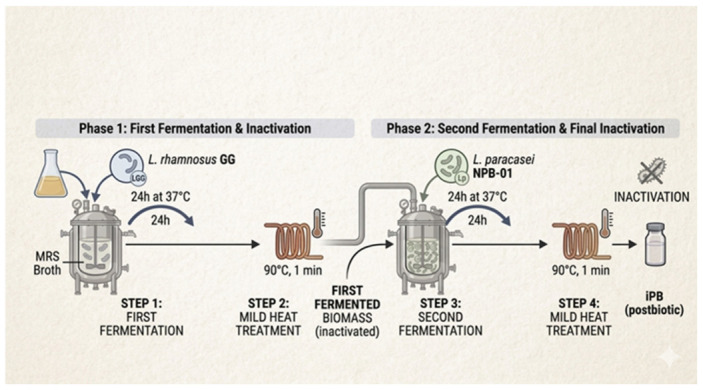
The sequential fermentation process for postbiotic production.

The third postbiotic product was obtained from the sequential fermentation process using *L. rhamnosus* GG and *L. paracasei* NPB-01 (iPB). Briefly, after the first fermentation of *L. rhamnosus* GG in MRS broth for 24 h at 37 °C, a mild heat treatment (90 °C, 1 min) was applied for inactivating the microbial population. Then, a second fermentation on MRS broth for 24 h at 37 °C was started by inoculating *L. paracasei* NPB-01 in the previous fermented biomass. Following the second fermentation process, a mild heat treatment (90 °C, 1 min) was performed (Figure 1). This sequential fermentation process was designed to overcome the typical limitations of traditional co-fermentation, such as inter-species competition for nutrients and microbial antagonism. In addition, by utilizing the heat-inactivated *L. rhamnosus* GG matrix as a pre-conditioned substrate for the second fermentation, it is possible to elicit a “metabolic priming” effect, allowing the second bacteria strain (*L. paracasei* NPB-01) to interact with and further transform the primary biomass of metabolites and other compounds produced by the first bacteria strain’s fermentation, potentially generating a more complex fingerprint of bioactive postbiotic compounds compared to the two single-strain preparations.

To evaluate the potential synergistic effect of the two strains without the sequential fermentation process, a physical mixture of Lpp and LGGp (Lpp + LGGp) was prepared by combining the two single-strain powders at their respective maximum doses immediately before cell stimulation. This combination (Lpp + LGGp) served as a control to distinguish the simple additive effect of the two strains from the metabolic signature generated by the sequential fermentation process (iPB).

All postbiotic preparations (LGGp, Lpp and iPB) investigated in this study were dried and were provided as food-grade powder by Science Power SrL (Milan, Italy).

A schematic representation of the sequential fermentation process is presented above. The diagram illustrates the production workflow: first, *L. rhamnosus* GG is fermented in a specific medium and then inactivated; subsequently, the resulting matrix is inoculated with *L. paracasei* NPB-01 for a second fermentation phase, followed by final inactivation and spray-drying to obtain the innovative postbiotic product (iPB).

### 2.2. Human Enterocyte Cell Line

For all experiments, we used the Caco-2 cell line (American Type Culture Collection, Middlesex, UK; accession number: HTB-37). Cells were grown in Dulbecco’s modified Eagle’s medium with a high glucose concentration (4.5 g/L) (Gibco, Berlin, Germany). The medium was supplemented with 10% fetal bovine serum, 1% l-glutamine, 1% non-essential amino acids, and 1% penicillin/streptomycin (Thermo Fisher Scientifics, Waltham, MA, USA). Cells were incubated at 37 °C and with 5% CO_2_. The medium was changed every 2 days.

### 2.3. Stimulation with Different Postbiotic Products

Caco-2 cells were seeded (0.1 × 10^6^ cells/well) in 12-well plates (Corning, Corning, NY, USA). After 15 days post-confluence, the cells were stimulated with the different postbiotic products for 48 h. Caco-2 cells stimulated with medium alone were used as controls. Experiments were performed 3 times and were carried out in triplicate.

### 2.4. Modulation of Epithelial Gut Barrier

The potential modulatory action on the epithelial gut barrier elicited by the study products was investigated using different tools, as previously adopted [14,15]. Enterocyte cell growth and differentiation were assessed using a proliferation assay and brush border enzyme lactase gene expression, respectively. The modulatory action exerted on mucous thickness was explored by evaluating mucin 2 (*Muc2*) gene expression. The effect on the tight junction network was explored by analyzing the gene expression of two major proteins, occludin and zonula occludens 1 (ZO-1).

Lastly, the potential modulatory action on innate immunity was explored by analyzing the production of the major innate immunity peptide, Human Beta-Defensin (HBD-2), by the enterocytes.

### 2.5. Cell Proliferation Assay

Human enterocyte proliferation assays were performed using MTT (the bromide salt of 3-(4,5-dimethylthiazol-2-yl)-2,5-diphenyl tetrazolium) (Sigma-Aldrich, Milan, Italy). Cells (104 cells/well) were seeded in 24-well plates (Corning, Inc., New York, NY, USA) with or without the postbiotic products at 37 °C in a humidified atmosphere containing 5% CO_2_. The cell viability was monitored by adding 5 mg/mL of MTT solution followed by 1 h incubation. The medium was then removed, and the converted dye was solubilized with acidic isopropanol (0.04–0.1 N HCl in absolute isopropanol). Absorbance was read at 570 nm using an Epoch Microplate Spectrophotometer (Bioteck, Winooski, VT, USA).

### 2.6. Real Time PCR

Total RNA from Caco-2 cells was extracted using TRIzol reagent (Gibco BRL, Paisley, UK). The reverse transcription to cDNA was performed with a High-Capacity RNA-to-cDNA™ kit (Life Technologies, Waltham, MA, USA) following the manufacturer’s instructions. The cDNAs were stored at −80 °C until analysis. Quantitative real-time PCR (qRT-PCR) analysis, carried out with TaqMan Gene Expression Master Mix (Applied Biosystems, Vilnius, Lithuania), was used to evaluate the gene expression of occludin (Hs05465837_g1) and ZO-1 (Hs01551871_m1). Gene expression of mucin 2 (Muc2) and lactase enzyme was evaluated using SYBR green Master Mix (Applied Biosystems, Grand Island, NY, USA). The primers used for Muc2 were forward (5′-CTCCGCATGAGTGTGAGT-3′) and reverse (5′-TAGCAGCCACACTTGTCTG-3′). The primers used for lactase were forward (5′-ACACGGTCGATTTCCTCTCT-3′) and reverse (5′-TGGGTTCTTCATGGTGGAGG-3′). The amplification protocol was 40 cycles of 15 s of denaturation at 95 °C, 60 s of annealing at 60 °C, and 60 s of elongation at 60 °C in a Light Cycler 7900HT (Applied Biosystems, Grand Island, NY, USA). Data were analyzed using the comparative threshold cycle method. We used the glucuronidase beta (GUS-B) gene to normalize the level of mRNA expression (TaqMan probes: Hs00939627_m1; SYBR green forward primer: 5′-GAAAATATGTGGTTGGAGAGCTCATT-3′; SYBR green reverse primer: 5′-CCGAGTGAAGATCCCCTTTTTA-3′). All primer sequences are summarized in Table 1.

### 2.7. Protein Levels of Tight Junction Markers Were Quantified by ELISA on Cell Lysates

At the end of the 48 h exposure period with the different postbiotic products or with medium alone, the cells were washed twice with cold PBS and lysed using RIPA buffer supplemented with protease inhibitors. Lysates were collected and centrifuged at 12,000× *g* for 10 min at 4 °C to remove cell debris. The supernatants were collected, and total protein concentration was determined using a Bradford assay. Equal amounts of total protein were used for the quantification of occludin, ZO-1 and MUC2 using commercially available ELISA kits (Elabscience Biotechnology Inc., Wuhan, China, detection limits are 0.1 ng/mL, 0.1 ng/mL and 0.19 ng/mL respectively) following the manufacturer’s instructions. Absorbance was measured at 450 nm using a microplate reader. Protein concentrations were calculated from standard curves and normalized to total protein content. Results were expressed as ng/mg of total protein.

### 2.8. Modulation of Innate Immunity

After stimulation with the postbiotics, cell supernatants were collected and stored at −80 °C until analysis. Production of HBD-2 was determined by an ELISA test using commercial enzymatic kits (Human DEFβ2/DEFB2, Elabscience Biotechnology Inc., Wuhan, China, detection limit of 62.5 pg/mL).

### 2.9. Statistical Analysis

All experiments were performed three times in triplicate. All data were collected in a dedicated database and analyzed by a statistician using GraphPad Prism 9.3.0 (La Jolla, CA, USA). Statistical analysis was carried out using one-way ANOVA followed by Tukey’s post hoc test. Differences were considered statistically significant at *p* < 0.05. The Kolmogorov–Smirnov test was used to determine whether variables were normally distributed. Considering the normal distribution, data were presented as mean ± standard deviation (SD).

## 3. Results

### 3.1. Modulation of Human Enterocyte Cell Growth and Differentiation

**Figure 2:**
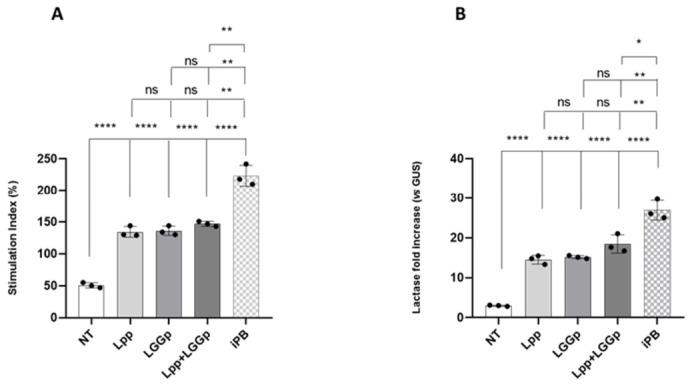
Effects on cell proliferation (stimulation index) (**A**) and cell differentiation (lactase expression fold increase) (**B**) elicited by the different postbiotic products on human enterocytes. After 15 days post-confluence, Caco-2 cells were stimulated for 48 h with 1 mg/mL *Lacticaseibacillus paracasei* NPB-01 postbiotic (Lpp), with 0.1 mg/mL *Lacticaseibacillus rhamnosus* GG postbiotic (LGGp), and with 1 mg/mL postbiotic product obtained by sequential fermentation of the two probiotic stains (iPB). Lpp + LGGp is the physical mixture of the two single-strain postbiotics. The stimulation index was evaluated by an MTT test, while the lactase fold increase was evaluated by real-time PCR. Statistical significance between specific conditions is indicated by brackets. The tiered arrangement, from bottom to top, represents sequential comparisons against NT, Lpp, LGGp, and Lpp + LGGp. Statistical significance was determined by one-way ANOVA with Tukey’s post hoc test; * *p* < 0.05, ** *p* < 0.01, **** *p* < 0.0001 vs. control., ns = no significance.

In preliminary dose–response and time course experiments, we defined the best experimental conditions for each postbiotic product. As shown in Figure 2A,B, both products obtained through the single-strain fermentation process (Lpp and LGGp) promoted the growth and differentiation of human enterocytes. No further increase in cell growth and differentiation was observed after adding Lpp and LGGp simultaneously at the maximum respective doses. On the contrary, the modulatory actions elicited by the postbiotic product derived from sequential fermentation (iPB) were significantly higher for both parameters compared with those obtained by the two single-strain postbiotic products alone or in combination.

### 3.2. Modulation of Tight Junction Proteins and Mucous Production in Human Enterocytes

**Figure 3:**
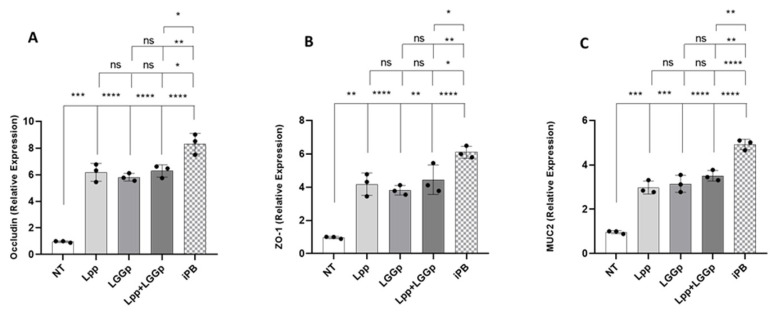
Effects on tight junction proteins occludin (**A**) and Zonulin-1 (ZO-1) (**B**) and on mucin2 (MUC2) (**C**) elicited by postbiotic products. After 15 days post-confluence, Caco-2 cells were stimulated for 48 h with 1 mg/mL *Lacticaseibacillus paracasei* NPB-01 postbiotic (Lpp), with 0.1 mg/mL *Lacticaseibacillus rhamnosus* GG postbiotic (LGGp), and with 1 mg/mL postbiotic product obtained by sequential fermentation of the two probiotics (iPB). Lpp + LGGp is the physical mixture of the two single-strain postbiotics. The concentration of occludin, ZO-1, and MUC2 was evaluated by real-time PCR. Statistical significance between specific conditions is indicated by brackets. The tiered arrangement, from bottom to top, represents sequential comparisons against NT, Lpp, LGGp, and Lpp + LGGp. Statistical significance was determined by one-way ANOVA with Tukey’s post hoc test; * *p* < 0.05, ** *p* < 0.01, *** *p* < 0.001, **** *p* < 0.0001 vs. control, ns = no significance.

**Figure 4:**
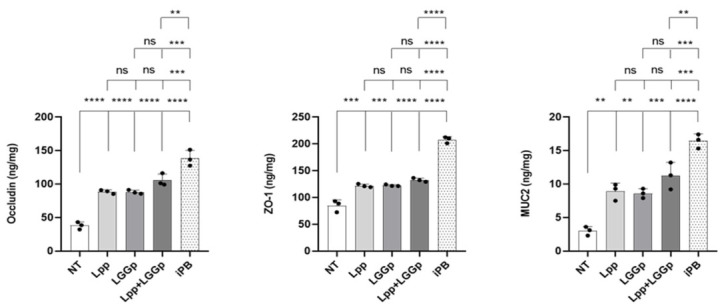
Protein expression of tight junction and mucus markers in Caco-2 cells following postbiotic treatments. Protein levels of ZO-1, occludin, and MUC2 were quantified by ELISA in cell lysates of Caco-2 cells after treatment with different postbiotics. All treatments resulted in increased protein levels compared to untreated controls, with the sequential postbiotic (iPB) showing the strongest effect. Data are expressed as mean ± SD and normalized to total protein content. Statistical significance between specific conditions is indicated by brackets. The tiered arrangement, from bottom to top, represents sequential comparisons against NT, Lpp, LGGp, and Lpp + LGGp. Statistical significance was determined by one-way ANOVA with Tukey’s post hoc test; ** *p* < 0.01, *** *p* < 0.001, **** *p* < 0.0001 vs. control, ns = no significance.

As shown in Figure 3, the incubation with all three postbiotic products led to an increase in the expression of the two major tight junction proteins, occludin and ZO-1 (Figure 3A,B). Also, the expression of the epithelial mucus layer protein MUC2 was modulated by these two postbiotic products (Figure 3C). No further stimulatory action for all three parameters was observed when adding Lpp and LGGp simultaneously at the maximum respective dose. On the contrary, iPB consistently induced a significantly greater stimulatory effect on all biomarkers compared to the other postbiotics alone or in combination. Protein quantification by ELISA confirmed the gene expression data, showing a significant increase in ZO-1 and occludin levels in treated cells compared to controls, suggesting improved epithelial barrier integrity (Figure 4).

### 3.3. Modulation of Innate Immunity

**Figure 5:**
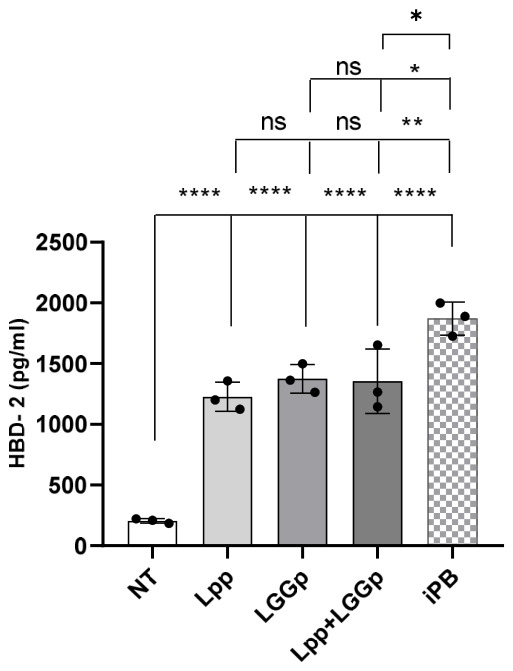
Effect of the postbiotic products on the innate immunity peptide HBD-2. After 15 days post-confluence, Caco-2 cells were stimulated for 48 h with 1 mg/mL *Lacticaseibacillus paracasei* NPB-01 postbiotic (Lpp), with 0.1 mg/mL *Lacticaseibacillus rhamnosus* GG postbiotic (LGGp), and with 1 mg/mL postbiotic product obtained by sequential fermentation of the two probiotics (iPB). The concentration of HBD-2, as a marker of innate immunity, was evaluated immunoenzymatically. Statistical significance between specific conditions is indicated by brackets. The tiered arrangement, from bottom to top, represents sequential comparisons against NT, Lpp, LGGp, and Lpp + LGGp. Statistical significance was determined by one-way ANOVA with Tukey’s post hoc test; * *p* < 0.05, ** *p* < 0.01, **** *p* < 0.0001 vs. control, ns = no significance.

To investigate the potential modulatory action elicited by the postbiotic products evaluated in this study, we measured the production of the major innate immunity peptide HBD-2 by human enterocytes stimulated with the study products for 48 h. As shown in Figure 5, the incubation with all the three postbiotic products led to an increase in the production of HBD-2 by human enterocytes. No further stimulatory action in HBD-2 production was observed when stimulating the cells simultaneously with Lpp and LGGp at the maximum respective dose. On the contrary, exposing human enterocytes to iPB resulted in a significantly higher stimulatory effect on HBD-2 production compared with that observed stimulating the cells with Lpp or with LGGp alone or in combination.

## 4. Discussion

Fermentation is a primary and essential process for producing postbiotics [16], it can significantly affect the composition and function of these products [17,18,19,20]. Several studies have highlighted the beneficial effects of different postbiotic products derived from single or multiple bacterial strains, either co-fermented or mixed post-fermentation [21,22,23]. In this study, we provided the first evidence of the beneficial actions on human enterocytes elicited by an innovative postbiotic (iPB) produced through sequential fermentation of two probiotic strains (i.e., *L. rhamnosus* GG and *L. paracasei* NPB-01). Our findings demonstrated that iPB exerts a more potent regulatory action on human enterocyte proliferation and differentiation, as demonstrated by MTT and brush border enzyme lactase increases, respectively. A parallel more potent effect was also observed on epithelial gut barrier architecture, as suggested by increased expression of the two major tight-junction proteins, occludin and ZO-1. We also found that iPB exposure resulted in a higher increase in the expression of Muc2 and the innate immunity peptide HBD-2 by human enterocytes. Muc2 is a key mucin protein forming the protective mucus layer in the intestine that is essential for epithelial gut barrier function [24]. The Muc2 mucus layer is essential in protecting the host not only against pathogens but also against the occurrence of allergies, limiting the exposure to environmental allergens [25]. In addition, by stimulating the production of key regulatory factors such as IL-10, Transforming Growth Factor-β (TGF-β), Retinoic Acid (RA), and thymic stromal lymphopoietin (TSLP) by epithelial and dendritic cells (DCs), Muc2 promotes the expansion of Treg cells and reduces inflammation, thereby restoring immune tolerance and homeostasis [25]

Beyond their structural and metabolic roles, intestinal epithelial cells actively participate in mucosal immunity by producing antimicrobial peptides like HBD-2. This small, cationic, antimicrobial peptide “farms” the microbiome, influencing its composition and preventing dysbiosis, which can lead to inflammatory conditions. HBD-2 possesses a broad-spectrum activity against bacteria, viruses, fungi and some parasites. However, in addition, HBD-2 activates a variety of immune cells and regulates cytokine/chemokine production, cell migration, proliferation, differentiation, angiogenesis, the wound healing process and maintenance of the epithelial barrier function. Alterations in the level of HBD-2 have been associated with the initiation and development of various inflammatory and allergic diseases [26,27]. Thus, this molecule could be considered as a cornerstone of mucosal innate immune system, providing a first line of defense against infections, dysbiosis, inflammation and allergic sensitization [14,28]. The higher modulatory activity elicited by superior induction of HBD-2 observed with iPB in our study suggests a potent activation of the innate sensing machinery. In this context, the role of intracellular receptors like NOD2 (Nucleotide-binding Oligomerization Domain-containing protein 2) is of particular relevance. NOD2 acts as a key sensor for bacterial peptidoglycan fragments, such as muramyl dipeptide, which are essential components of postbiotic matrices. As recently reported, the NOD2 signaling pathway is a fundamental driver for the secretion of antimicrobial peptides and the maintenance of intestinal homeostasis in response to beneficial *Lacticaseibacillus* strains [29]. Therefore, it is plausible that the sequential fermentation process optimizes the release of specific Microbe-Associated Molecular Patterns (MAMPs) that more effectively trigger the NOD2-HBD-2 axis compared to standard postbiotic preparations.

For all variables investigated into the study, we found a higher modulatory action elicited by iPB compared with that obtained by single postbiotics alone or in combination, suggesting that the sequential fermentation process likely induces a unique metabolic signature. We hypothesize that the first fermentation step with LGG modifies the substrate and releases primary metabolites that, following heat inactivation, serve as a specialized matrix for the subsequent fermentation by *L. paracasei* NBP01. This ‘metabolic priming’ may trigger the synthesis of a more complex array of bioactive compounds, such as specific peptides or metabolites. This study introduces the concept of sequential postbiotic design as a strategy to maximize the health-promoting potential of lactic acid bacteria.

While these results are promising, some limitations should be acknowledged. First, this study focused on two specific probiotic strains, both individually and in a sequential combination; therefore, the reproducibility and generalizability of these effects to other bacterial taxa remain to be established. Additionally, the sequential fermentation was performed exclusively by inoculating LGG followed by *L. paracasei* NBP01. Future research exploring a reversed inoculation order or different timing intervals could provide deeper insights into how microbial succession shapes the final postbiotic activity.

Furthermore, it is important to consider that the specific order of strain inoculation in a sequential fermentation process can significantly influence the final metabolic profile. Since the first microorganism modifies substrate availability and produces metabolites that affect the growth of the second strain, the sequence used in this study (*L. rhamnosus* GG followed by *L. paracasei* NPB-01) was chosen based on preliminary data. However, investigating a ‘reverse’ fermentation—where *L. paracasei* NPB-01 is inoculated first—could provide further insights into how microbial succession shapes the postbiotic properties. Future studies will be aimed at exploring this alternative sequence and different fermentation timings to further optimize the health-promoting potential of the resulting product.

Although the biological impact of iPB is highlighted by the results of this study, the specific effector molecules—whether they be secreted metabolites, cell wall components (such as the capsule), or genomic DNA fragments— as well as the respective mechanisms of action, have yet to be fully characterized. It has been suggested that many postbiotics’ effects involve the activation of toll-like receptors and related intracellular pathways, such as NF-κB signaling [28]. Thus, the results of this study suggest that the sequential fermentation optimizes the concentration of ‘Microbe-Associated Molecular Patterns’ (MAMPs) or secreted factors that are recognized by TLRs on human cells [30]. This enhanced modulatory action on the epithelial gut barrier and innate immunity suggest that iPB could provide a more efficient protective effect against conditions characterized by alterations in intestinal permeability, dysbiosis, immune tolerance and inflammation. Nevertheless, our findings highlight the potential of sequential fermentation as a novel and superior biotechnological platform for postbiotic production. This approach could pave the way for a new generation of functional ingredients with enhanced efficacy in modulating human health. Further investigations are warranted to test different strain combinations and to broaden the evaluation of intestinal barrier integrity, systemic immunity, and cellular differentiation pathways.

## 5. Conclusions

In conclusion, our data support the use of sequential fermentation to produce a high-performance postbiotic. iPB stands out as a promising candidate for functional food applications and clinical interventions aimed at strengthening the intestinal barrier and immunity. Future studies involving ‘omics’ technologies (metabolomics and proteomics) will be essential to pinpoint the exact molecular effectors responsible for these observed synergistic benefits.

## Figures and Tables

**Table 1:** Primer sequences used for qRT-PCR analysis.

Target Gene	Forward	Revers
***Muc2***	5′-CTCCGCATGAGTGTGAGT-3′	5′-TAGCAGCCACACTTGTCTG-3′
***Lactase***	5′-ACACGGTCGATTTCCTCTCT-3′	5′-TGGGTTCTTCATGGTGGAGG-3′
***Gus-B***	GAAAATATGTGGTTGGAGAGCTCATT	CCGAGTGAAGATCCCCTTTTTA

## Data Availability

The original contributions presented in this study are included in the article. Further inquiries can be directed to the corresponding author.

## Abbreviations

The following abbreviations are used in this manuscript:

iPB	Innovative postbiotic
ZO-1	Zonula occludens-1
MUC-2	Mucin-2
HBD-2	Beta-defensin 2
LGG	*Lactocticaseibacillus* GG
LGGp	*Lactocticaseibacillus* GG postbiotic
Lpp	*Lactocticaseibacillus paracasei* NPB-01 postbiotic
qRT-PCR	Quantitative real-time PCR
GUS-B	Glucuronidase beta
TGF-β	Transforming growth factor-β
RA	Retinoic acid
TSLP	Thymic stromal lymphopoietin
DCs	Dendritic cells
